# Barriers to Oral Health Care in Children: Determinants of Dental Neglect

**DOI:** 10.3390/children13050621

**Published:** 2026-04-30

**Authors:** Andreea Mihaela Kiș, Dan Iovanescu, Liana Todor, Ramona Amina Popovici, Laria-Maria Trusculescu, Dana Emanuela Pitic, Andreea Salcudean, Adina Feher, Andrada Ioana Dumitru, Porumb Anca, Iustin Olariu

**Affiliations:** 1 Department I of Management and Communication in Dental Medicine, Faculty of Dental Medicine, Victor Babes University of Medicine and Pharmacy of Timisoara, 300041 Timisoara, Romania; kis.andreea@umft.ro (A.M.K.); laria.trusculescu@umft.ro (L.-M.T.); 2 Doctoral School, Victor Babes University of Medicine and Pharmacy of Timisoara, 300041 Timisoara, Romania; dan.iovanescu@umft.ro (D.I.); dana.pitic@umft.ro (D.E.P.); adina.feher@umft.ro (A.F.); andrada.dumitru@umft.ro (A.I.D.); 3 Department of ENT, Victor Babes University of Medicine and Pharmacy of Timisoara, Eftimie Murgu Square No. 2, 300041 Timisoara, Romania; 4 Faculty of Medicine and Pharmacy, Department of Dentistry, University of Oradea, 410087 Oradea, Romania; 5 Department of Bioethics, Deontology and Medical Communication, George Emil Palade University of Medicine, Pharmacy, Science, and Technology of Targu Mures, 540142 Targu Mures, Romania; andreea.salcudean@umfst.ro; 6 Department of Dental Medicine, Faculty of Medicine and Pharmacy, University of Oradea, 410087 Oradea, Romania; anca.porumb@uoradea.ro; 7 Department of Dentistry, Faculty of Dentistry, Vasile Goldiş Western University of Arad, 94-96 Revoluţiei Blvd., 310025 Arad, Romania; olariu.iustin@uvvg.ro

**Keywords:** children, oral health, prevention, health barriers

## Abstract

**Highlights:**

**What are the main findings?**

- Family socio-economic factors matter: income and parental education are significantly associated with children’s daily tooth-brushing habits, while lower-income families face financial/logistical barriers to dental visits.
- Parental education was not significantly linked to annual dental check-ups, though a trend suggests some influence; reasons for avoiding check-ups differ by income (cost vs. time/perceived need).

**What are the implications of the main findings?**

- Children’s oral health is strongly shaped by social, economic, and educational context, meaning medical care alone is not enough to address the problem.
- Effective improvement requires integrated public policies (education, accessible services, financial support), especially targeting vulnerable and low-income families.

**Abstract:**

**Background/Objectives**: Neglect of children’s oral health is a major concern at international, national, and regional levels. Of all the health problems that can occur in childhood, dental ones are among the most common. Tooth decay, for example, is a chronic condition in children and can have long-term consequences, especially in otorhinolaryngology and pediatric diseases if not treated properly. **Methods**: The data collection method was questionnaire. Questionnaires were administered to parents regarding oral hygiene habits and access to dental services; data were collected in dental offices across Timiș County, encompassing urban, peri-urban, and rural settings. Children enrolled in the study underwent clinical dental examinations to assess their oral health status (dental caries, gingival diseases, developmental anomalies). **Results**: Parental education level was not significantly associated with the habit of annual dental check-ups (χ^2^, *p* = 0.092); however, a directional trend was observed. Total monthly family income was significantly associated with the stated reason for not attending dental check-ups (one-way ANOVA, *p* = 0.043): families with lower incomes more frequently cited financial and logistical barriers, whereas higher-income families cited lack of time or perceived lack of necessity. Parental education level (*p* < 0.001) and family income (*p* < 0.001) were both significantly associated with daily tooth-brushing frequency. **Conclusions**: The efforts of specialists must be increased through coherent policies, adapted education, and real support for vulnerable groups. An informed child, with supported parents, is a child with a real chance at a healthy life. This is not just a professional opinion, but a collective responsibility.

## 1. Introduction

Oral neglect of children is a major concern at international, national, and regional levels. International health organizations, such as the World Health Organization, emphasize the importance of prevention and early treatment of dental diseases [1]. At the national level, public health policies seek to ensure equal access to medical care, including dental care, for all children. At the regional level, demographic and socio-economic characteristics influence access to dental services. Pediatric oral health is essential for the overall well-being of children and for their harmonious development [2]. Of all the health problems that can occur in childhood, dental ones are among the most widespread. Tooth decay, for example, is one of the most common chronic conditions in children and can have long-term consequences, especially in otorhinolaryngology and pediatric diseases if not treated properly [3]. Therefore, ensuring adequate access to quality dental care and promoting prevention are crucial for maintaining children’s oral health. Pediatric oral health focuses not only on the treatment of dental diseases, but also on education and prevention to ensure optimal oral health throughout life. In childhood, baby teeth play a critical role in the correct development of mastication, speech, weight, growth, self-esteem, communication, and facial aesthetics [4,5]. Untreated dental problems during this period can affect the correct alignment of permanent teeth and can lead to more severe ear, nose, and throat (ENT) complications in the future; therefore, oral health plays a key role in the development early childhood illnesses. Healthy teeth contribute to the correct formation of sounds, the articulation of words and the development of communication skills [6]. Oral problems, such as untreated caries, premature loss of baby teeth or malocclusion, and mouth breathing can affect the development of language and speech [3,6]. When the child’s teeth are affected, the pronunciation of sounds can become unclear, which leads to communication difficulties [7,8]. Studies have shown that poor oral health can contribute to the occurrence of cardiovascular diseases, diabetes, and respiratory infections [9,10,11]. Therefore, preventive dental care is essential not only for oral health, but also for the general health of children. The socio-economic context plays a crucial role in children’s dental health. Therefore, families with low incomes and limited access to medical services are often the most affected by dental problems [12]. Lack of education on oral hygiene, reduced access to dental care products, and financial barriers to treatments are factors that contribute to the increased incidence of dental problems in these communities [13]. It is essential that public health policies address without discrimination and ensure equal access to quality dental care for all children, regardless of their socio-economic background. Education and awareness are fundamental pillars for improving pediatric dental health [14]. Untreated dental infections can have serious systemic consequences. For example, bacteria from the oral cavity can enter the bloodstream and cause infections in other parts of the body, such as the heart (bacterial endocarditis) or joints (septic arthritis) [15]. Dental infections can also worsen pre-existing medical conditions, such as diabetes, and weaken the child’s immune system. In extreme cases, severe dental infections can be life-threatening, thus emphasizing the importance of timely preventive and curative interventions [16].

Causes of dental infections include untreated dental caries that allow bacteria to enter the tooth pulp; gum disease (gingivitis or periodontitis) that weakens the supporting tissues; dental trauma such as cracks or fractures that create an entry point for bacteria; incomplete or incorrect dental treatments, including defective fillings; and poor oral hygiene that promotes the accumulation of bacterial plaque [17]. The symptoms of a dental infection are primarily an intense, throbbing toothache; increased sensitivity to extreme temperatures (hot/cold); swelling of the face or gums; redness and inflammation in the affected area; bad breath or an unpleasant taste in the mouth; and fever and general malaise in severe cases [18]. If an abscess is left untreated, the bacteria can spread to other areas of the body, causing serious complications, such as extensive infections of the jawbone (osteomyelitis), maxillary sinusitis, septicemia, and an increased risk of cardiovascular disease [15,19]. The elected treatment in almost all cases comprises antibiotics prescribed to combat the infection, along with anti-inflammatory analgesics to alleviate pain and reduce inflammation.

This study aims to identify risk factors contributing to dental neglect, including economic, cultural, and parental education barriers, with a view to characterizing the main obstacles to access to appropriate dental services for children. Additionally, the study also highlights the importance of integrating developmental insights with epidemiological data and preventive strategies in the collaborative work of dentists, pediatricians, and otorhinolaryngologists.

## 2. Materials and Methods

### 2.1. Study Design and Participants

This study was a multicenter, observational, descriptive, and analytical investigation. It was performed between January 2024 and September 2025 at a private practice in Timișoara in collaboration with two private dental and pediatric practices located in peri-urban and urban areas. Applied to a sample of 257 parents of children showing signs of dental neglect, all data were anonymized and stored on encrypted, password-protected servers accessible only to study personnel. The target population studied comprised children aged between 3 and 12 years, coming from different socio-economic backgrounds, who had been exposed to neglect in dental medical care.

### 2.2. Data Collection

Data were collected using a structured questionnaire administered to parents regarding oral hygiene habits and access to dental services. Children enrolled in the study underwent clinical dental examinations to assess their oral health status (dental caries, gingival diseases, developmental anomalies). All data were collected after obtaining written informed consent from parents or legal guardians. Semi-structured interviews were also conducted with participating dentists; ear, nose, and throat (ENT) specialists; and pediatricians to identify the most common problems encountered in children with dental neglect and to obtain information about the history of dental and general practice visits.

We first conducted a descriptive analysis of the demographic profile of the children and parents who participated in the study, which focused on the access to and attitudes toward pediatric dental care. The following variables were analyzed: child age and sex, respondent relationship to child, living environment, family situation, family size, and average income per adult member and per total member (including children).

For the purposes of this study, dental neglect was operationally defined as the failure to seek timely and appropriate preventive or curative dental care for a child, as evidenced by at least one of the following criteria: (a) absence of a dental check-up in the preceding 12 months despite the presence of a dental condition; (b) presence of untreated dental caries, periodontal disease, or developmental dental anomaly confirmed on clinical examination; or (c) referral from a pediatrician or otorhinolaryngologist citing oral health neglect. This composite operational definition is consistent with the framework proposed by the American Academy of Pediatric Dentistry (AAPD). Inclusion criteria were (1) age 3–12 years; (2) meeting at least one criterion of the above operational definition of dental neglect; (3) willingness of the child’s parent or legal guardian to provide written informed consent and to participate in the questionnaire. Exclusion criteria were (1) presence of a chronic systemic condition known to independently affect oral health (e.g., severe diabetes mellitus, autoimmune disorders, rare genetic syndromes); (2) receipt of dental treatment within the preceding six months that could confound clinical assessment; (3) refusal of informed consent; (4) inability to cooperate with clinical examination due to severe disability; (5) evidence of regular dental surveillance without signs of neglect.

From 257 assessed children, 175 (68.1%) were ultimately included; 30 lacked parental consent, 10 could not cooperate, and 42 had received dental treatment within the preceding six months.

### 2.3. Ethical Considerations

The current study was evaluated and approved by the Scientific Research Ethics Committee of Victor Babeș University of Medicine and Pharmacy, Timișoara Approval No. 109/16.12.2022 rev. 2025 in accordance with the Declaration of Helsinki. Written informed consent was obtained from all caregivers before enrollment, emphasizing confidentiality and voluntary participation from parents or legal guardians, and assent from children aged ≥3 years to 12 in accordance with the Declaration of Helsinki and GDPR.

### 2.4. Statistical Analysis

The collected data were statistically analyzed to highlight the correlations between dental neglect and oral health problems. The tools used for data collection were Microsoft Word 365 applications for designing questionnaires and Microsoft Excel 365 for collecting and structuring data. The data were analyzed, interpreted, and correlated using IBM SPSS v.27.0 software. The following statistical tests were used to analyze the data obtained: descriptive statistical analysis, including frequency distributions, percentages, and mean and standard deviation; chi-square test (χ^2^) for independence, to evaluate the association between categorical variables; one-way ANOVA, to compare group means; Pearson correlation coefficient, to analyze linear relationships between numerical variables; independent samples *t*-test (where necessary); and Fisher’s exact test for contingency tables with small expected frequencies. Specifically, one-way ANOVA was applied to examine differences in total monthly family income across the five categories of the stated reason for not attending dental check-ups, with income as the dependent variable and reason as the grouping factor. The Pearson correlation coefficient was used to analyze the linear relationship between numerical variables; the independent sample *t*-test (when necessary) was applied to compare means between two groups; and Fisher’s exact test was used for contingency tables with reduced frequencies. The statistical significance threshold used was *p* < 0.05.

## 3. Results

The age distribution of the children shows that the majority of the surveyed children (73 children, representing 41.7%) were in the 6–8 age group; another 50 children (28.3%) were in the 3–5 age category, and 52 children (30.0%) were between 9 and 12 years old (Table 1).

The most represented age group was 6–8 years (41.7%). With regard to sex distribution, boys showed a higher prevalence of oral health problems and suboptimal tooth-brushing habits compared to girls. The majority of questionnaire respondents were mothers (125; 71.7%), followed by fathers (38; 21.7%) and other relatives (12; 6.6%), reflecting the predominant role of mothers in supervising children’s health behaviors, particularly in contexts involving pain or fear. The majority of children resided in urban environments. The most frequent family configuration comprised two children per household. Regarding family situation, 143 children (81.7%) lived with both parents, 29 (16.7%) were raised by a single parent, and 3 cases (1.6%) were classified as “other situation”. Only 68 families (38.9%) reported a parental educational attainment above secondary school level.

### 3.1. Income-Level Influence

**Figure 1:**
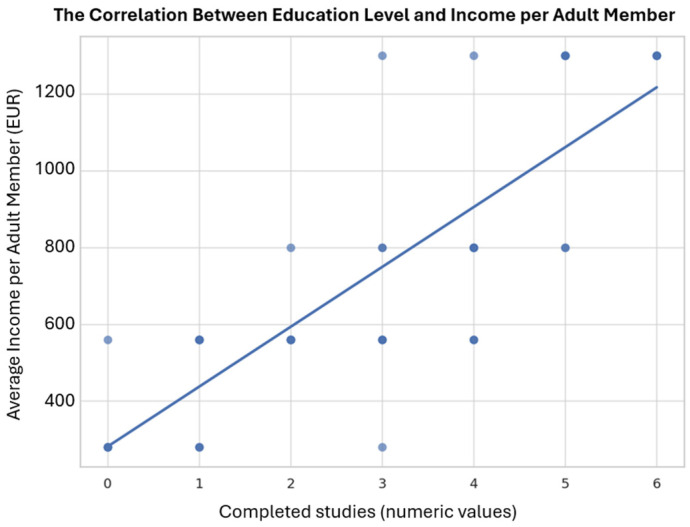
Correlation between education level and income/adult member. Education level was coded as an ordinal variable (0 = no formal education to 6 = postgraduate) and treated as pseudo-continuous for visualization purposes. Due to non-normal distribution of variables (Shapiro–Wilk test, *p* < 0.05), Spearman’s rho is reported as the primary correlation measure, while Pearson’s r is shown for comparison.

The total monthly income of the participating families varies significantly, with values ranging between 550.24 Euro and 5109.36 Euro. The arithmetic mean of income is 1868.06 Euro, with a median value of 1572.11 Euro. The first quarter (25%) of the distribution is below 1100.48 Euro, while 75% of the families have a total monthly income below 2358.17 Euro. These data suggest a considerable dispersion of financial resources, also reflected by the standard deviation of 1296.40 Euro (Table 2). A significant relationship was observed between the educational level of the parents and the average monthly income per adult member of the family (Figure 1). Given the non-normal distribution of both variables (Shapiro–Wilk test, *p* < 0.05), Spearman’s rank correlation coefficient was computed as the primary measure: ρ = 0.856 (*p* < 0.001), indicating a very strong positive monotonic association. Pearson’s r = 0.840 is reported for reference. This strong correlation highlights the importance of education as a predictive factor for the socio-economic conditions of families, suggesting that children from more educated households benefit from greater financial resources and, consequently, greater capacity to access dental care. 

### 3.2. Socio-Residential Factors

**Figure 2:**
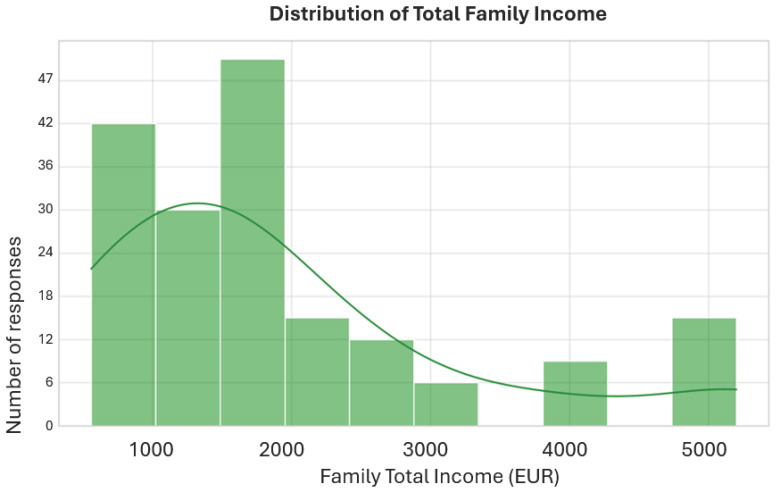
Distribution of total family income.

**Figure 3:**
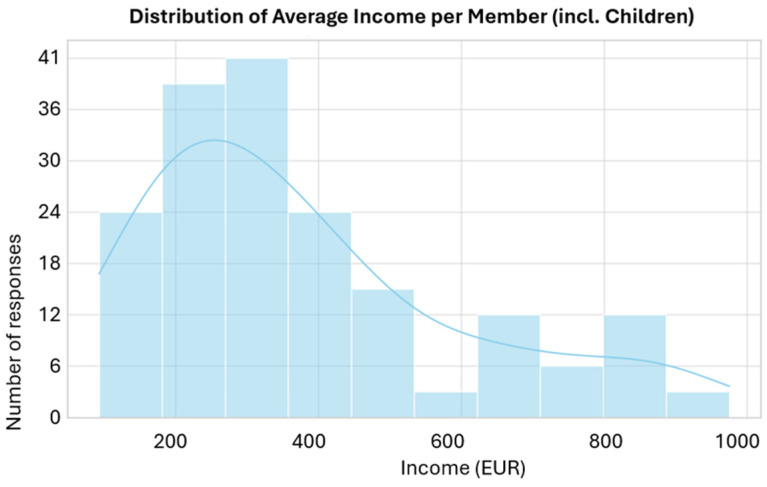
Distribution of average income per member including children.

The majority of respondents live in privately owned housing (34 people, 56.7%). Another 18 families (30%) live in rented housing, and 7 respondents (11.7%) stated that they live in social housing. Only one case was classified as “Other”. These data reflect a relatively high degree of housing stability within the sample (Figure 2 and Figure 3).

To assess the relationship between housing type and total family income, we used a numerical coding of the category “housing”. The Pearson correlation coefficient between the two variables is r = 0.19, indicating a weak positive correlation. This result suggests that higher income is slightly associated with home ownership, but this relationship is not strong. However, the median income distribution differs by housing type, as highlighted in the boxplot diagram below. To assess the relationship between living environment (urban vs. rural) and housing type (personal property, rented, social, other), the chi-square test for independence was used. The contingency table shows that in rural areas, 13 people live in their own homes, 7 rent, 5 live in social housing, and 1 lives in another type of housing. In urban areas, 21 people live in personal property, 11 rent, and 2 live in social housing. The chi-square test indicated a *p*-value of 0.255, which means that there is nostatistically significant correlation between living environment and housing type (at a significance level of 0.05). To explore the relationship between family situation (both parents, single parent, other) and housing type, the same chi-square test was used.

As shown in Table 2, the most frequently reported average income per adult member was 550.80 EUR, corresponding to 73 families (41.71%). The minimum income tier (275.40 EUR/adult member) was reported by 32 families (18.29%), while 70 families (40.0%) reported an intermediate or higher income level. The average income per family member (including children) shows a wider distribution (mean = 525.33 EUR), reflecting the impact of family size on per capita resources (Figure 3).

### 3.3. Household Utilities

Analysis of access to utilities according to the living environment was descriptive, which comprised inferential analysis of access to utilities among respondent families and an assessment of the correlations between living environment (urban or rural) and their availability. The utilities included gas, electricity, running water, heating system, internet, and car.

Of the 175 respondent families, 85 (48.3%) declared that they have gas, while 90 (51.7%) do not have access to this utility. All 175 respondent families declared that they have electricity.

The chi-square test applied to evaluate the relationship between living environment and access to gas generated a *p*-value = 0.5781. This value does not indicate a statistically significant correlation, so access to this utility does not differ significantly between the living environments.

**Figure 4:**
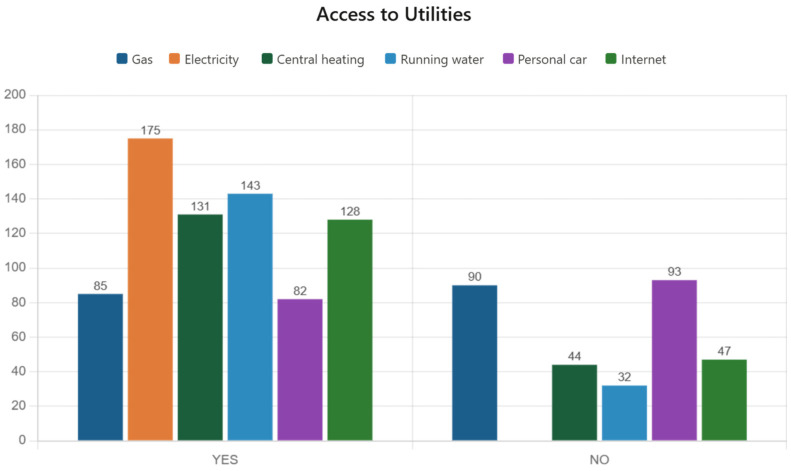
Access to utilities.

Of the 175 respondent families, 131 (75.0%) declared that they have central or gas heating, while 44 (25.0%) do not have access to this utility. The chi-square test applied to evaluate the relationship between living environment and access to heating generated a *p*-value = 0.0026. This value indicates a statistically significant correlation, which means that access to this utility varies depending on the living environment (urban or rural). This finding does not mean that those families do not have heating, but rather that it is provided from other sources and not from gas or central heating (Figure 4).

Of the 175 respondent families, 143 (81.7%) declared that they have water, while 32 (18.3%) do not have access to this utility. The chi-square test applied to evaluate the relationship between living environment and access to water generated a *p*-value = 0.0001. This value indicates a statistically significant correlation, which means that access to this utility varies depending on the living environment (urban or rural). Considering that the drinking water network is less widespread in rural areas and that dug or drilled wells are common there, we can conclude that 100% of respondents have access to drinking water.

Of the 175 respondent families, 82 (46.7%) declared that they have a car, while 93 (53.3%) do not have access to this utility. The chi-square test applied to evaluate the relationship between living environment and access to a car generated a *p*-value of 1.0000. This value does notindicate a statistically significant correlation, so access to this utility does not differ significantly between living environments. This resource weighs heavily in the results of the study regarding visits to dental offices.

Of the 175 respondent families, 128 (73.3%) declared that they have internet access, while 47 (26.7%) do not have access to this utility. The chi-square test applied to evaluate the relationship between living environment and internet access generated a *p*-value = 0.3560. This value does notindicate a statistically significant correlation, so access to this utility does not differ significantly between rural and urban living environments.

### 3.4. Daily Brushing Habits

Daily brushing habits were analyzed based on the number of times per day that parents reported brushing their teeth. The distribution shows that 50 respondents (28.6%) do not brush their teeth at all, 79 (45.1%) brush once a day, and 46 (26.3%) maintain a twice-daily brushing habit. The overall mean is 0.98 brushings/day, with a standard deviation of 0.75. These data indicate a poor practice of daily oral hygiene among the respondents.

The chi-square test applied to examine the relationship between living environment and daily brushing habit generated a *p*-value = 0.663. This value does not indicate a statistically significant correlation, suggesting that living environment (urban or rural) does not significantly influence the frequency of daily tooth brushing.

**Figure 5:**
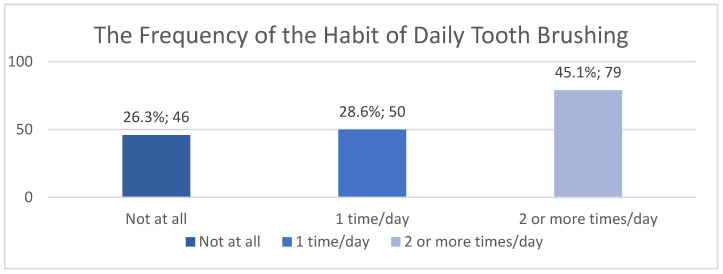
Analysis of daily brushing habits and associated factors.

The analysis of the relationship between family situation and brushing habits returned a *p*-value of 0.727. Again, there is no statistically significant association, indicating that family structure (both parents or single parent) does not clearly influence daily oral hygiene behavior (Figure 5).

In contrast, the analysis based on parental education level yielded a *p*-value of 0.00008, indicating a statistically significant correlation. This suggests that a higher level of education is associated with healthier daily oral hygiene habits. Also, the ANOVA analysis that tested the differences between the total income of the families according to the frequency of brushing revealed a *p*-value = 0.000003. This correlation is significant, which indicates that the family income is associated with better daily oral hygiene. Families with higher incomes tend to adopt more rigorous practices regarding personal oral hygiene.

### 3.5. Analysis of the Habit of Annual Dental Check-Ups and Associated Correlations

The analysis of the habit of performing an annual dental check-up revealed that only 82 of the 175 respondents (46.7%) stated that they go to the dentist annually, while 93 respondents (53.3%) do not have this habit. The binary mean of the responses is 0.47, which indicates a deficient practice regarding annual prophylactic checks. The chi-square test applied to assess the association between living environment and annual dental check-up generated a *p*-value of 0.848, which is not statistically significant. This suggests that living in an urban or rural area does not significantly influence the probability of having an annual check-up. For family situation, the *p*-value obtained was 0.560, also indicating the absence of a statistically significant relationship between family structure and the habit of visiting the dental office annually.

Parental education level was not significantly associated with the habit of annual dental check-ups (χ^2^, *p* = 0.092); however, a directional trend was observed, with more educated parents showing higher compliance with annual dental visits. This result does not support a formal inferential conclusion but is noted for its exploratory relevance (Cramér’s V = 0.426, indicating a substantial effect size despite non-significance at α = 0.05).

The ANOVA test that evaluated the differences between total family income and annual check-up habit generated a *p*-value = 0.001718, indicating a statistically significant correlation. This means that families with higher incomes are significantly more likely to have regular dental check-ups.

The association between the frequency of daily brushing and annual check-ups was also analyzed. The *p*-value obtained from the chi-square test is 0.000457, indicating a statistically significant correlation. Therefore, there is a significant relationship between adequate oral hygiene behavior and compliance with annual dental visits—those who brush twice a day are more likely to monitor their oral health through regular check-ups.

**Figure 6:**
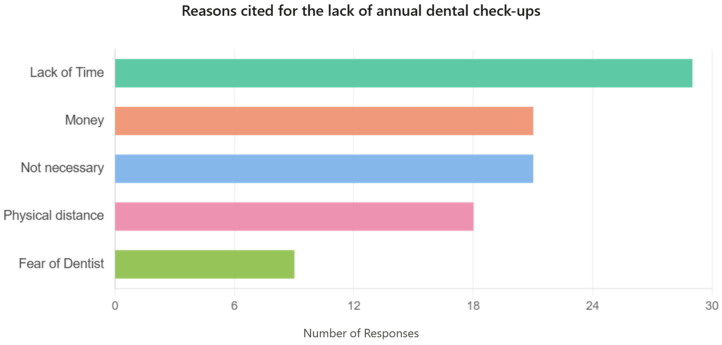
Reasons for the lack of annual dental check-ups.

Of the total number of respondents who stated that they do not have an annual dental check-up, only a subset of 93 people also provided a reason. Responses such as “Not applicable” were excluded from the analysis, considered undecided or irrelevant for the purpose of this analysis. The distribution of valid reasons is as follows: 26 respondents (28.0%) cited lack of time, 20 (21.5%) indicated costs, 20 (21.5%) did not consider an annual check-up necessary, 18 (19.35%) mentioned the distance from the office, and 9 (9.65%) stated that they were afraid (Figure 6).

The chi-square test applied to the reasons for lack of control and living environment generated a *p*-value = 0.0037, indicating a statistically significant correlation. Therefore, the type of reason given for not having annual dental check-ups is influenced by living environment—certain reasons are more common in rural areas than in urban areas, and vice versa.

The results of the ANOVA test indicated a *p*-value = 0.04260, statistically significant, suggesting that the total monthly family income influences the stated reason for not having dental check-ups. Families with lower incomes are more likely to invoke financial or logistical barriers, while families with higher incomes tend to justify the absence of check-ups by lack of time or personal perception considerations.

Table 3 provides a comprehensive synthesis of all inferential statistical analyses performed in this study, including chi-square statistics, degrees of freedom, *p*-values, and Cramér’s V effect size estimates for all bivariate associations involving categorical variables, and Cohen’s d for the income × annual check-up comparison. Effect size interpretation follows the thresholds proposed by Cohen (1988): V < 0.10 negligible; V 0.10–0.19 small; V 0.20–0.39 moderate; V ≥ 0.40 large; d ≥ 0.80 large [20].

## 4. Discussion

Children’s oral health is an essential indicator of overall health and quality of life. Despite advances in health education and access to services, the prevalence of dental caries in children remains high in many regions, including Romania [21]. The purpose of this study is to provide insight into the socio-demographic and behavioral factors associated with dental neglect among children. These results highlight the central role that economic, social, and educational factors play in shaping oral health behavior. The literature strongly supports the need for early and integrated interventions to reduce inequalities and improve oral health. In this context, dental neglect in children—often associated with insufficient parental or caregiver involvement—remains a significant public health concern [22]. Pediatric dental health requires an interdisciplinary approach, involving collaboration between dentists, pediatricians, otorhinolaryngologists, educators, and parents, with each playing a critical role in ensuring appropriate dental care and promoting oral health [23]. The strong association between parental education and family income reflects the link between education level and socio-economic status. Thus, children from more advantaged families tend to prioritize dental health. These results are consistent with conceptual frameworks on oral health inequities that emphasize that education remains a fundamental determinant of health-related behaviors and access to resources. Prevention plays a central role in maintaining children’s oral health. By educating parents and children about the importance of oral hygiene and by implementing preventive measures such as dental sealants and fluoride applications, the risk of cavities and other dental diseases can be significantly reduced [24]. Even with a preventive program there are still many barriers that can prevent children from accessing appropriate dental care. These include economic barriers, such as the high cost of dental treatments, which can be prohibitive for many families. Geographical barriers, such as long distances to the nearest dental office, can also limit access to care. Cultural and educational barriers are also significant, as lack of knowledge about the importance of oral health and fear of the dentist can discourage families from seeking preventive care for their children [25]. It is essential to address these barriers through public health policies and education programs to improve access to dental care for all children [26]. Family income emerged as a significant factor in the practice of preventive dentistry, with children from families with above-average incomes more likely to attend annual dental check-ups, while families with lower incomes reported lower rates of attendance. Unfortunately, the high treatment cost represents for many children in Romania the main barrier to adequate and timely dental treatments. Many families cannot afford dental services due to high prices, especially for complex treatments. In many countries, dental services are not fully covered by public insurance, and private insurance may be inaccessible. In our country, at the level of the constitution and Law 95/2006 on health reform, medical treatments of any kind are free according to the law, but in practice they are very expensive and more than 96% of dental clinics are private [27,28]. Financial resources continue to play a key role in facilitating access to preventive dental services. Furthermore, low-income families were more likely to report structural barriers such as cost and distance, while those with higher incomes more frequently cited lack of time or need. Some families do not prioritize dental care due to lack of time, resources, or misconceptions. Lack of knowledge about the importance of oral hygiene and regular visits to the dentist can lead to neglect of dental care. In some cultures, there are myths and misconceptions about dental health, such as the belief that baby teeth are not important because they will be replaced by permanent teeth [29,30]. Although no significant associations were found between urban–rural residence and frequency of tooth brushing or attendance at annual dental check-ups, the place of residence was significantly associated with the type of barriers reported, suggesting that while overall behaviors may not differ substantially, the underlying constraints influencing these behaviors are context-dependent. This supports the interpretation that access to care is shaped not only by individual behavior, but also by structural and geographic factors. In rural or remote areas, access to a dentist is difficult, as the distance to dental offices is long and transportation can be a problem. There are areas where the number of specialists is very small and waiting lists are long; in addition, in many places, emergency dental treatments are not available 24/7, and dental offices are overloaded and cannot provide timely appointments for all patients. Also, parents’ work schedules may interfere with the ability to take children to the dentist during office hours. Lack of flexibility in scheduling consultations and the lack of dental services outside of standard business hours can hinder access to dental care for children [31,32]. Furthermore, the healthcare system is excessively bureaucratic; therefore, obtaining a free or reimbursed consultation can be difficult due to administrative formalities, insufficient funding of public dental services (in many health systems, funds for dentistry are limited and free services are limited), and long waiting times; even when treatments are free, children can have to wait months for an appointment [33,34]. Also, in immigrant families or minority groups, there may be a reluctance to visit dentists for linguistic or cultural reasons [35,36]. Addressing these barriers requires joint effort from authorities, dentists, medical caregivers, parents, and the community to ensure equal access to dental care for all children. 

To overcome these barriers, an integrated and multidimensional approach is needed. Public health policies should include measures to finance dental treatment for low-income families and support dental prevention programs in schools and communities [37,38]. The long-term consequences of oral neglect encompass impairments in speech articulation and masticatory function. For correct phonation, children require healthy primary teeth to allow optimal movement of the tongue, lips, and jaw; dental deficiencies are specifically associated with difficulties in pronouncing fricative and alveolar consonants (e.g., /t/, /d/, /s/, /z/, /n/, /L/) [39]. The direct consequence of untreated caries is premature primary tooth loss, which disrupts the eruption sequence of permanent teeth and may necessitate costly orthodontic or restorative interventions in the future. Progressing infections may cause dental abscesses affecting adjacent structures [40]. Overall, the findings emphasize that dental neglect is strongly influenced by socio-economic inequalities and behavioral patterns, rather than by single demographic factors alone. The results support a multifactorial interpretation, in which financial resources, educational background, and health-related behaviors interact to shape access to and utilization of dental care. First, dental offices must be reintroduced into schools. Second, it is necessary to stimulate parental participation through community campaigns, digital media, and collaborations with pediatricians, otorhinolaryngologists, and general practitioner doctors. Financial support, as well as the provision of free check-ups, should be assumed public policies, not just recommendations. The literature supports that outreach initiatives and parental education have proven effects on reducing caries and increasing regular visits [41]. Education must be accompanied by infrastructure: mobile offices, screening programs, and training teachers to recognize signs of dental disease. In the long term, the economic benefits of prevention far outweigh the costs of late intervention [42]. Oral health education programs, carried out in schools and communities, can significantly contribute to informing children and parents about the importance of oral hygiene and regular visits to the dentist. Encouraging oral hygiene practices from an early age can prevent cavities and other dental problems, contributing to the formation of healthy habits that will last a lifetime [43].

## 5. Limitations

This study has several limitations. First, the sample includes only children meeting the operational criteria for dental neglect and does not include a general population comparator or control group. This limits the ability to draw comparative conclusions about the relative strength of the identified determinants. Future research should address this by adopting a case-control design, including children without dental neglect recruited from the same clinical settings (e.g., those attending routine preventive care).

Second, the study does not include standardized clinical oral health indices (such as DMFT/dmft, PUFA/pufa, or the Basic Periodontal Examination), which limits the ability to directly link socio-demographic and behavioral factors to oral health outcomes. This is because clinical data were collected as part of routine practice, and the presence of conditions such as caries, gingivitis, or developmental anomalies was used as inclusion criteria rather than as primary outcome measures.

## 6. Conclusions

The data analyzed confirms a known but often ignored reality: a child’s oral health is deeply dependent on the social, educational, and economic context of the family. We are at a moment of opportunity: parents are interested, but support mechanisms are lacking. If we want a generation of children with healthy teeth, we must act now, through coherent policies, effective education, and accessible services. Resolving this situation requires an integrated approach. Oral prevention in pediatric dentistry is not only a medical objective, but also an indicator of social justice. The efforts of specialists must be increased through coherent policies, adapted education, and real support for vulnerable groups. An informed child, with supported parents, is a child with a real chance at a healthy life. This is not just a professional opinion, but a collective responsibility. Based on the above conclusions, economically adapted solutions can fundamentally transform the approach to this problem. The following strategic directions should be formulated to improve children’s oral health: reintroducing dental offices in schools, especially in rural and disadvantaged communities; developing and distributing information materials adapted to the educational and cultural level of families; financing free annual check-ups for all children under 14 and encouraging preventive treatments; and continuous monitoring of oral health indicators through digital registries at county and community level.

## Figures and Tables

**Table 1:** Patients’ demographics.

Variable	Category	No. (n)	Percent (%)
Child age	3–5 years	50	28.3%
	6–8 years	73	41.7%
	9–12 years	52	30.0%
Child sex	Boy	91	52.0%
	Girl	84	48.0%
Parent respondent	Mother	125	71.7%
	Father	38	21.7%
	Other relative	12	6.6%
Environment	Urban	99	56.7%
	Rural	76	43.3%
Family status	Both parents	143	81.7%
	Single parent	29	16.7%
	Different situation	3	1.6%
Number of children/family	1 child	32	18.3%
	2 children	82	46.7%
	3 children	41	23.3%
	4 children	20	11.7%
Education level	No studies	23	13.33%
	Primary school	32	18.33%
	Gymnasium	26	15.00%
	High school	26	15.00%
	Post-secondary studies	29	16.67%
	University studies	23	13.33%
	Postgraduate studies	16	8.34%

**Table 2:** Distribution of average incomes of participating families.

Income	Reported Value (Euro)	No. (n)/Percent (%)	Observations/Interpretation
Average income per adult member	275.40	32 (18.29%)	Minimum reported level
	550.80	73 (41.71%)	Most common value
	786.86	32/(18.29%)	Intermediate level
	1278.65	38/(21.71%)	Maximum reported level
Total range	275.40–1278.86		Mean = 777.13 Euro
Average income per family member (incl. children)	91.67	-	Minimum observed value
	183.54	20	
	275.40	26	Most common value
	393.43	15	
	958.99	-	Maximum observed value
Total range	91.67–958.99		Mean = 525.33 Euro; Wide distribution, significant socio-economic variation

**Table 3:** Summary of inferential statistical results with effect size measures (Cramér’s V and Cohen’s d). N = 175 (full sample); n = 32 for the subset analysis of barriers to check-up attendance. * *p* < 0.05; ns = not significant.

Independent Variable	Dependent Variable	Test	df	*p*	Sig.	Effect Size	Interpretation
Education level	Daily brushing	χ^2^	12	<0.001	*	V = 0.574	Large
Daily brushing frequency	Annual check-up	χ^2^	2	0.001	*	V = 0.506	Large
Education level	Annual check-up	χ^2^	6	0.092	ns	V = 0.426	Large (ns)
Urban/rural residence	Barrier type (subset n = 32)	χ^2^	4	0.004	*	V = 0.696	Very large
Total family income (RON)	Annual check-up (Yes/No)	Independent *t*-test	58	0.002	*	d = 0.851	Large
Total family income (RON)	Barrier type (5 groups)	One-way ANOVA	4, 27	0.043	*	η^2^ = SPSS	Moderate

## Data Availability

The data used and/or analyzed during the current study are available on request from the corresponding author due to confidentiality, ethical considerations, and all applicable regulations regarding sensitive or personal information.

## References

[1] World Health Organization (2022). Global Oral Health Status Report: Towards Universal Health Coverage for Oral Health by 2030.

[2] Han S.Y., Chang C.L., Wang Y.L., Wang C.S., Lee W.J., Vo T.T.T., Chen Y.L., Cheng C.Y., Lee I.T. (2025). A Narrative Review on Advancing Pediatric Oral Health: Comprehensive Strategies for the Prevention and Management of Dental Challenges in Children. Children.

[3] Predescu I.-A., Kiș A.M., Pitic D.E., Anton A., Dinu Ș., Păcurar M., Bud E., Popovici R.A., Popa M., Olariu I. (2024). Mouth Breathing Syndrome—An Interdisciplinary Approach. Rom. J. Oral Rehabil..

[4] Foster K.D., Woda A., Peyron M.A. (2006). Effect of texture of plastic and elastic model foods on the parameters of mastication. J. Neurophysiol..

[5] Sheiham A. (2006). Dental caries affects body weight, growth and quality of life in pre-school children. Br. Dent. J..

[6] Budală D.G., Lupu C.I., Vasluianu R.I., Ioanid N., Butnaru O.M., Baciu E.R. (2023). A Contemporary Review of Clinical Factors Involved in Speech-Perspectives from a Prosthodontist Point of View. Medicina.

[7] Bommangoudar J.S., Chandrashekhar S., Shetty S., Sidral S. (2020). Pedodontist’s Role in Managing Speech Impairments Due to Structural Imperfections and Oral Habits: A Literature Review. Int. J. Clin. Pediatr. Dent..

[8] Martinelli L., Fornaro E.F., Oliveira C.J., Ferreira L.M.D.B., Rehder M.I.B.C. (2011). Correlations between speech disorders, mouth breathing, dentition and occlusion. Rev. CEFAC.

[9] Monteiro V.R., Brescovici S.M., Delgado S.E. (2009). The occurrence of lisp in eight- to 11-year-old children from municipal schools. Rev. Soc. Bras. Fonoaudiol..

[10] Leng Y., Hu Q., Ling Q., Yao X., Liu M., Chen J., Yan Z., Dai Q. (2023). Periodontal disease is associated with the risk of cardiovascular disease independent of sex: A meta-analysis. Front. Cardiovasc. Med..

[11] Păunică I., Giurgiu M., Dumitriu A.S., Păunică S., Pantea Stoian A.M., Martu M.A., Serafinceanu C. (2023). The Bidirectional Relationship between Periodontal Disease and Diabetes Mellitus-A Review. Diagnostics.

[12] Marusiak M.J., Paulden M., Ohinmaa A. (2023). Professional oral health care prevents mouth-lung infection in long-term care homes: A systematic review. Can. J. Dent. Hyg..

[13] de Silva-Sanigorski A.M., Calache H., Gussy M., Dashper S., Gibson J., Waters E. (2010). The VicGeneration study—A birth cohort to examine the environmental, behavioural and biological predictors of early childhood caries: Background, aims and methods. BMC Public Health.

[14] Lewis C., Cantrell D., Domoto P. (2004). Oral health in the pediatric practice setting: A survey of Washington state pediatricians. Pediatrics.

[15] Lewis C., Boulter S., Keels M.A., Krol D.M., Mouradian W.E., O’Connor K.G., Quinonez R.B. (2009). Oral health and pediatricians: Results of a national survey. Acad. Pediatr..

[16] Foster H., Fitzgerald J. (2005). Dental disease in children with chronic illness. Arch. Dis. Child..

[17] Teal L., Sheller B., Susarla H.K. (2024). Pediatric Odontogenic Infections. Oral Maxillofac. Surg. Clin. N. Am..

[18] Siqueira J.F., Rôças I.N. (2022). Treatment of Endodontic Infections.

[19] Tavares W.L., Brito E.H., de Oliveira M.M., Figueiredo L.C., Faveri M., Mayer M.P.A., de Figueiredo J.A.P., Duarte P.M., Feres M., Cortelli S.C. (2012). Clinical and microbiological analysis of acute dental abscesses. Oral Surg. Oral Med. Oral Pathol. Oral Radiol..

[20] Cohen J. (1988). Statistical Power Analysis for the Behavioral Sciences.

[21] Dumitru A.I., Kis A.M., Badea M.-A., Lacatusu A., Boia M. (2025). Health-Related Quality of Life and Internalising Symptoms in Romanian Children with Congenital Cardiac Malformations: A Single-Centre Cross-Sectional Analysis. Healthcare.

[22] Seni A.-G., Sălcudean A., Popovici R.A., Olariu I., Cincu M.-G., Jinga V., Trusculescu L.-M., Pitic D.E., Cosoroabă R.M., Kis A. (2025). The Prevalence of Dental Caries Among Children Aged 6–11: A Cross-Sectional Study from Mureș County, Romania. Medicina.

[23] Petersen P.E., Kwan S. (2011). Equity, social determinants and public health programmes--the case of oral health. Community Dent. Oral Epidemiol..

[24] Gaffar B., Farooqi F.A., Nazir M.A., Bakhurji E., Al-Khalifa K.S., Alhareky M., Virtanen J.I. (2022). Oral health-related interdisciplinary practices among healthcare professionals in Saudi Arabia: Does integrated care exist?. BMC Oral Health.

[25] Colegiul Medicilor Stomatologi din România (CMSR) (2024). Barometrul Sănătății Orale—Studiu Național Asupra Sănătății Oro-Dentare a Românilor, a Comportamentului și Nevoilor Identificate la Nivelul Populației în Privința Sănătății Orale. Direcții de Intervenție și Propuneri Pentru Îmbunătățirea Politicilor Publice și a Programelor Naționale de Sănătate Orală—Raport Final.

[26] Chisini L.A., Costa F.D.S., Demarco G.T., da Silveira E.R., Demarco F.F. (2021). COVID-19 pandemic impact on paediatric dentistry treatments in the Brazilian Public Health System. Int. J. Paediatr. Dent..

[27] Quiñonez C., Figueiredo R., Locker D. (2015). Public preferences for expanding government-funded dental care in Canada. J. Public. Health Dent..

[28] (2006). Law No. 95/2006 on Healthcare Reform.

[29] OECD/European Observatory on Health Systems and Policies (2023). Romania: Country Health Profile 2023.

[30] Hilton I.V., Stritzel G. (2006). Unmet dental needs and barriers to care for children and adults with significant special health care needs. Spec. Care Dent..

[31] Gao X., Hamzah S.H., Yiu C.K.Y., McGrath C., King N.M. (2021). Dental fear and anxiety in children and adolescents: Qualitative study using YouTube. J. Med. Internet Res..

[32] Zhao Y., Surdu S., Langelier M. (2023). Parental Perspectives on Barriers to Pediatric Oral Health Care: Associations with Children’s and Families’ Characteristics. Pediatr. Dent..

[33] Ribeiro G.L., Gomes M.C., de Lima K.C., Martins C.C., Paiva S.M., Granville-Garcia A.F. (2015). Work absenteeism by parents because of oral conditions in preschool children. Int. Dent. J..

[34] Sălcudean A., Truşculescu L.M., Popovici R.A., Şerb N., Paşca C., Bodo C.R., Crăciun R.E., Olariu I. (2024). Dental anxiety—A psychosocial cause affecting the quality of life—A systematic review. Rom. J. Oral Rehab.

[35] Peres M.A., Macpherson L.M.D., Weyant R.J., Daly B., Venturelli R., Mathur M.R., Listl S., Celeste R.K., Guarnizo-Herreño C.C., Kearns C. (2020). Oral diseases: A global public health challenge. Lancet.

[36] Nelson S., Slusar M.B., Albert J.M., Riedy C.A. (2017). Do baby teeth really matter? Changing parental perception and increasing dental care utilization for young children. Contemp. Clin. Trials..

[37] Tiwari T., Randall C.L., Cohen L.K., Newton J.T., Farsides B., Watt R.G., Williams D.M., Listl S., Kearns A., Petersen P.E. (2020). A case for the integration of social and behavioral sciences in oral health interventions. J. Public Health Dent..

[38] Dye B.A., Tan S., Smith V., Lewis B.G., Barker L.K., Thornton-Evans G., Eke P., Beltrán-Aguilar E.D., Horowitz A.M., Li C.-H. (2007). Trends in oral health status: United States, 1988–1994 and 1999–2004. Vital Health Stat..

[39] Mathu-Muju K.R., McLeod J., Donnelly L. (2022). The impact of a school-based dental program on children’s oral health and well-being. J. Can. Dent. Assoc..

[40] Watt S., Dyer T.A., Marshman Z., Jones K. (2024). Does poor oral health impact on young children’s development? A rapid review. Br. Dent. J..

[41] Dumitrescu A.L. (2024). Dental Appliances and Oral Hygiene: Tips and Recommendations.

[42] Watt R.G., Harnett R., Daly B., Fuller S.S., Kay E., Morgan A., Munday P., Nowjack-Raymer R., Treasure E.T. (2006). Evaluating oral health promotion: Need for quality outcome measures. Community Dent. Oral Epidemiol..

[43] Griffin S.O., Barker L.K., Griffin P.M., Cleveland J.L., Kohn W. (2009). Oral health needs among adults in the United States with chronic diseases. J. Am. Dent. Assoc..

